# M1-like tumor-associated macrophages cascade a mesenchymal/stem-like phenotype of oral squamous cell carcinoma via the IL6/Stat3/THBS1 feedback loop

**DOI:** 10.1186/s13046-021-02222-z

**Published:** 2022-01-06

**Authors:** Yuanhe You, Zhuowei Tian, Zhong Du, Kailiu Wu, Guisong Xu, Meilu Dai, Yan’an Wang, Meng Xiao

**Affiliations:** 1 Department of Oral and Maxillofacial-Head and Neck Oncology, Shanghai Ninth People’s Hospital, School of Medicine, Shanghai Jiao Tong University, Shanghai, China; 2 College of Stomatology, Shanghai Jiao Tong University, Shanghai, China; 3 National Center for Stomatology; National Clinical Research Center for Oral Disease, Shanghai, China; 4 Shanghai Key Laboratory of Stomatology and Shanghai Research Institute of Stomatology, Shanghai, China; 5 Department of Orthodontics, Shanghai Stomatological Hospital & School of Stomatology, and Shanghai Key Laboratory of Craniomaxillofacial Development and Diseases, Fudan University, Shanghai, China

**Keywords:** Macrophage, Oral squamous cell carcinoma, Epithelial-mesenchymal transfer, Cancer-stem like cells, Interleukin 6

## Abstract

**Background:**

Tumor-associated macrophages (TAMs) have a leading position in the tumor microenvironment. Previously, we have demonstrated that M1-like TAMs activated by exosome-transferred THBS1 promote malignant migration in oral squamous cell carcinoma (OSCC). However, the functional roles and associated molecular mechanisms of the activated M1-like TAMs need to be further clarified in OSCC.

**Methods:**

Conditioned Media (CM) were harvested from the exosome activated M1-like TAMs. We measured the malignant behaviors of OSCC under the treatment of CM from M1-like TAMs by performing colony forming assays, invasion assays, wound-healing assays, spheroid forming assays and in *vivo* xenograft experiments. The underlying mechanisms were investigated by RNA-seq, cytokines analysis, intracellular signaling pathway analysis, ChIP assays, bioinformatics analysis and validation.

**Results:**

M1-like TAMs significantly promoted the epithelial-mesenchymal transition (EMT) process, and induced the cancer-stem like cells (CSCs) by upregulating the expression of MME and MMP14 in OSCC cells. Cytokine analysis revealed a shark increase of IL6 secretion from M1-like TAMs. Blocking IL6 in the CM from M1-like TAMs could significantly weaken its effects on the colony forming, invasion, migration, microsphere forming and xenograft forming abilities of OSCC cells. Cellular signaling assays indicated the activation of Jak/Stat3 pathway in the OSCC cells treated by the CM from M1-like TAMs. Blocking the activation of the Jak/Stat3 pathway could significantly weaken the effects of M1-like TAMs on the colony forming, invasion, migration, microsphere forming and xenograft forming abilities of OSCC cells. Further RNA-seq analysis and bioinformatics analysis revealed an increased expression of THBS1 in the OSCC cells treated by M1-like TAMs. Bioinformatics prediction and ChIP assays revealed the activation of Stat3 by CM from M1-like TAMs could directly promote the transcription of THBS1 in OSCC cells.

**Conclusions:**

We proposed that M1-like TAMs could cascade a mesenchymal/stem-like phenotype of OSCC via the IL6/Stat3/THBS1 feedback loop. A better understanding on the functional roles and associated molecular mechanisms of M1-like TAMs might facilitate the development of novel therapies for supplementing the current treatment strategies for OSCC patients.

**Supplementary Information:**

The online version contains supplementary material available at 10.1186/s13046-021-02222-z.

## Background

Tumor-associated macrophages (TAMs) are key components of the tumor microenvironment (TME) in solid tumors [1]. TAMs can promote tumor progression at different levels by promoting genetic instability, nurturing cancer stem cells, supporting metastasis, and taming protective adaptive-immunity [2, 3]. A growing body of evidence has suggested that TAMs polarization is more complex than the M1 and M2 binary classification [4]. The existence of diverse TAMs subsets has been investigated according to their polarization requirement, phenotype, and function [5, 6]. Therefore, polarized TAMs are gradually described as M1-like or M2-like macrophages. In addition, the conclusions that M2-like TAMs are associated with unfavorable survival and that M1-like TAMs are associated with favorable outcomes are being questioned in some cancers [7–9].

Recently, the existence and roles of M1-like TAMs have been identified and revealed. High infiltration of M1-like TAMs has been identified to be associated with aggressive features in some cancers [7, 9, 10]. Previously, we demonstrated that exosome-transferred THBS1 polarized macrophages to the M1-like phenotype through p38, Akt, and SAPK/JNK signaling at the early phase in oral squamous cell carcinoma (OSCC) [8]. Comparatively, M1-like TAMs were not observed in normal oral mucosa or oral leukoplakia (precancerous lesion) [8]. The infiltration and polarization of M1-like TAMs might be an essential event during the carcinogenesis and progression of OSCC.

Oral squamous cell carcinoma, a major component of head and neck squamous cell carcinoma (HNSCC), remains one of the most lethal cancers worldwide [11–13]. To date, no definitive driver mutation has been identified in OSCC or HNSCC. Therefore, increasing attention has been given to the TME in the initiation and progression of OSCC [13, 14]. Macrophages play a central role in the TME, and our previous study demonstrated that M1-like TAMs could promote the malignant migration in OSCC [8]. In this study, we aimed to investigate in detail the underlying mechanisms, by which the activated M1-like TAMs regulate the malignant progression of OSCC.

## Methods

### Cell culture, conditioned media preparation and exosome harvest

Oral squamous cell carcinoma cell lines SCC25 and Cal27 were obtained from American Type Culture Collection (ATCC) and cultured as previously reported [8]. THP-1 cells were obtained from ATCC, and Peripheral blood mononuclear cells (PBMCs) were obtained from healthy controls [8]. THP-1 derived macrophages and PBMCs derived macrophages were differentiated and cultured as previously reported [8]. Exosomes were obtained from SCC25 cells, Cal27 cells and identified as previously reported, which were used to educate macrophages as previously reported [8].

### Colony forming assay

OSCC cells were suspended, counted, diluted and seeded at 1,000 cells/ well into 6-well plates and cultured for 7 days under different treatment. Cellular colonies were fixed, stained and compared as previously reported [15].

### Invasion assay

Transwell assays were performed to determine the invasion ability of OSCC cells under different treatment. As previously reported, 50 μl Matrigel (1:8 diluted, BD Bioscience) was coated on the chamber beforehand [16]. OSCC cells (5×10^4^ cells/ well) were suspended in 200μl of fresh media and plated into Millicell chambers (8 μm, Millipore Corporation) with 300μl of culture media containing 10% FBS and 300μl CM from exosome-treated macrophages in the bottom chambers [8]. Invaded cells were fixed, stained and compared as previously reported [16].

### Wound-healing assay

Wound-heal assays were performed to determine the migration ability of OSCC cells under different treatment. OSCC cells were seeded in 12-well plates and scratched by a sterile 200μl pipette tip when the cells reached 70-80% confluency. Cells were washed with 1x PBS twice to remove cellular debris and then cultured with serum-free media under different treatment. The width of the wound was photographed and measured at 1h and 24h

### Spheroid formation assay

Spheroid formation assays were performed to evaluate the regulation of cancer stem-like cells (CSCs). OSCC cells were dissociated and suspended in spheroid medium consisting of serum-free DMEM/F12 (Gibco, USA), human bFGF (20 ng/ml, Sino Biological Inc., China), human EGF (20 ng/ml, Sino Biological Inc., China), and B-27 supplement (Life Technologies, USA) in 6-well ultra-low attachment dishes (Corning, USA) under different treatment for 10 days [15]. The spheroid morphology was observed microscopically, and the number of spheroids was counted and compared.

### RNA-seq and data analysis

RNA-seq-based transcriptome profiling was performed for the OSCC cells under different treatment by Beijing Genomics Institute (Wuhan), using the BGISEQ-500 platform (Additional file 1A). After filtering, the sequencing data were mapped to the human reference genome hg38 (Assembly: GCF_000001405.38_GRCh38.p12) using HISAT and Bowtie2 software. The transcript quantification and normalization were performed using RSEM software package. The differentially expressed genes (DEGs) were identified by R package (Q-value≤0.001 and fold change>1.5). The DEGs were subjected to functional classification using GO and KEGG analysis.

### Cytokines analyses

Cytokine patterns were analyzed for the culture supernatants of macrophages under different treatment by using the Luminex^TM^xMAP technology using High Sensitivity 9-Plex Human ProcartaPlex^TM^ Panel (ThermoFisher Scientific, USA). All samples were run in triplicate as previously described [8].

#### Enzyme-linked Immunosorbent Assays (ELISA)

The levels of Interleukin 6 (IL6) secretion were validated and compared in the culture supernatants of macrophages under different treatment by ELISA according to the manufacturer’s instructions (eBioscience, USA). The concentration of THBS1 in the exosomes of OSCC cells was detected by ELISA according to the manufacturer’s instructions (Abcam, UK).

### Quantitative real-time PCR (qRT-PCR) assay

Total RNA was extracted and reversely transcribed using the PrimeScript RT reagent Kit (TaKaRa, Japan) according to the protocols recommended by the manufacturer. The cDNA was subjected to qRT-PCR detection using a SYBR Green Premix Kit (TaKaRa, Japan) [8]. The relative expression was calculated using the 2^-ΔΔCT^ method for the following genes: VIM, CDH2, MME, MMP14, IL6, CD68, CD80, CD86, and THBS1.

### Intracellular signaling pathway analysis

OSCC cells were lysed after treatment with CM from M1-like TAMs or M0 for 6h. Cell lysates were assayed using the Proteome Profiler Human Phospho-Kinase Array Kit according to the manufacturer’s protocol (R&D systems®, USA).

### Western blotting (WB)

For immunoblotting, cellular extracts were acquired by using RIPA Lysis Buffer containing proteinase inhibitor cocktail (Innovation, USA). After subjecting the lysates to SDS-PAGE electrophoresis, proteins were transferred onto a polyvinylidene difluoride membrane by electroblotting. The membranes were then blocked and incubated with primary antibodies (anti-phospho-Stat3 (Y705), anti-Stat3, anti-THBS1, anti-N-cadherin, anti-Vimentin, anti-CD10 and anti-MT1-MMP from Cell Signaling Technology, USA; anti-β-actin antibody, from BOSTER Biological Technology, China). Specific antibody-bound protein bands were detected with ECL Plus reagent (Millipore, USA) under Amersham Imager 600 (GE, USA) [8].

### Immunofluorescence assay

Exosome uptake by macrophages was examined as previously reported [8]. Differently treated OSCC cells were fixed, permeabilized and blocked as previously reported [15]. Then, the cells were incubated with anti-phospho-Stat3 (Y705) antibody (Cell Signaling Technology, USA) overnight at 4 °C followed by secondary antibody (Life, USA). Cellular nuclei were stained with DAPI (Roche, USA). Fluorescently labelled OSCC cells were examined using a ZEISS fluorescent imaging microscope (ZEISS, German).

### Chromatin immunoprecipitation (ChIP)

The chromatin immunoprecipitation assay was performed according to the instructions of the manufacturer (SimpleChIP® Enzymatic Chromatin IP Kit, Cell Signaling Technology, USA). The chromatin of treated OSCC cells was fixed, lysed, and sonicated. P-Stat3 antibody and IgG (Cell Signaling Technology, USA) was used to enrich the promoter regions of THBS1.The binding DNA fragments were analyzed by qPCR (primer sequences for THBS1: Forward: CAT TCC GGG AGA TCA GCT CG; Reverse: AAC TTC TCA GAA AAG TCG GTG CC).

### Xenograft experiments

Male BALB/c nude mice (nu/nu, aged 4-5 weeks and weighing approximately 20 g) were purchased from Shanghai Laboratory Animal Center (Shanghai, China) and were housed under specific-pathogen-free (SPF) conditions in the experimental animal care center of the Ninth People’s Hospital of the Shanghai Jiao Tong University School of Medicine [15]. Animal welfare and experimental procedures were conducted in compliance with the Guide for Care and Use of Laboratory Animals and the related ethical regulations of the hospital. The Animal Care and Use Committees of the hospital approved all experimental procedures.

Briefly, the nude mouse xenograft tumor models were established by subcutaneous injection of 5×10^6^ OSCC cells/per site. All the mice implanted with OSCC cells were randomly divided into the groups under different treatment (n=6 for each group). To evaluate the effects of CM from macrophages on the tumorigenesis of OSCC cells, mice with OSCC xenografts were received intraperitoneal injection of 1ml CM from M1-like TAMs or from M0 every other day, respectively. Ruxolitinib (Jak inhibitor, 50 mg/kg, i.p., every other day; Selleck, USA) was used to evaluate the effect of IL6/Stat3 signaling on the tumorigenesis of OSCC cells treated by CM from M1-like TAMs. Tumor volumes (length × width^2^/2) were monitored and compared [15].

### Immunohistochemical (IHC) Staining

Sections of 5 μm were prepared from paraffin-embedded samples. After deparaffinization, rehydration, and antigen retrieval, endogenous peroxidase activity was quenched. IHC staining was performed with primary antibody (mouse anti-human Ki67 antibody, mouse anti-human N-cadherin antibody, mouse anti-human Vimentin antibody mouse and anti-human THBS1 antibody from Santa Cruz, USA; rabbit anti-human p-Stat3 antibody from Cell Signaling Technology, USA) overnight at 4 °C. Then, the IHC staining was achieved by incubation with a biotinylated secondary antibody and staining with a DAB kit (GTVision, China). The immunoreactivity scores were recorded by multiplying the staining intensity and the percentage of positive cancer cells [16].

### Bioinformatics analysis and validation

Bioinformatics analysis was performed based on the TCGA HNSCC cohort. Only cases of primary HNSCC were filtered and included for further analysis of expression patterns of CD68, CD80, CD86, VIM, SNAI1, TWIST1, CDH2, MET, MME, MMP14, and IL6. Expression heat-maps of defined gene sets were generated and clustered online, and detailed data were downloaded for subsequent statistical analysis [8]. Besides, we used Spearman’s correlation analysis to describe the correlation between THBS1 and CD80, CD86, IL6, SNAI1, TWIST1, VIM, CDH2, MME or MMP14, respectively. A validated cohort was constructed based on 25 primary OSCC cases to investigate the expression correlation between IL6 and CD68, CD80 or CD86, respectively. Another validated cohort was constructed based on 40 primary OSCC cases to investigate the expression correlation between p-Stat3 and THBS1. The patients involved in this study signed written informed consent, and the study was approved by the Medical Ethics Committee of the Ninth People’s Hospital, Shanghai Jiao Tong University School of Medicine. The binding sites of p-STAT3 in promoters were predicted on JASPAR database (http://jaspar.genereg.net/) [17].

### Statistical analysis

All statistical analyses in this study were conducted with SPSS 20.0 software (SPSS, Inc., Chicago, IL, USA). Data are presented as the mean ± SD. The significant difference between two groups was determined by Student’s *t*-test. Crosstab analyses were performed for all the involved cases and Chi-squared tests were performed to assess the expression correlations between two variables [8]. A *p*-value <0.05 was considered statistically significant.

## Results

### Conditioned media from M1-like TAMs promote the malignant progression of OSCC cells

**Fig. 1:**
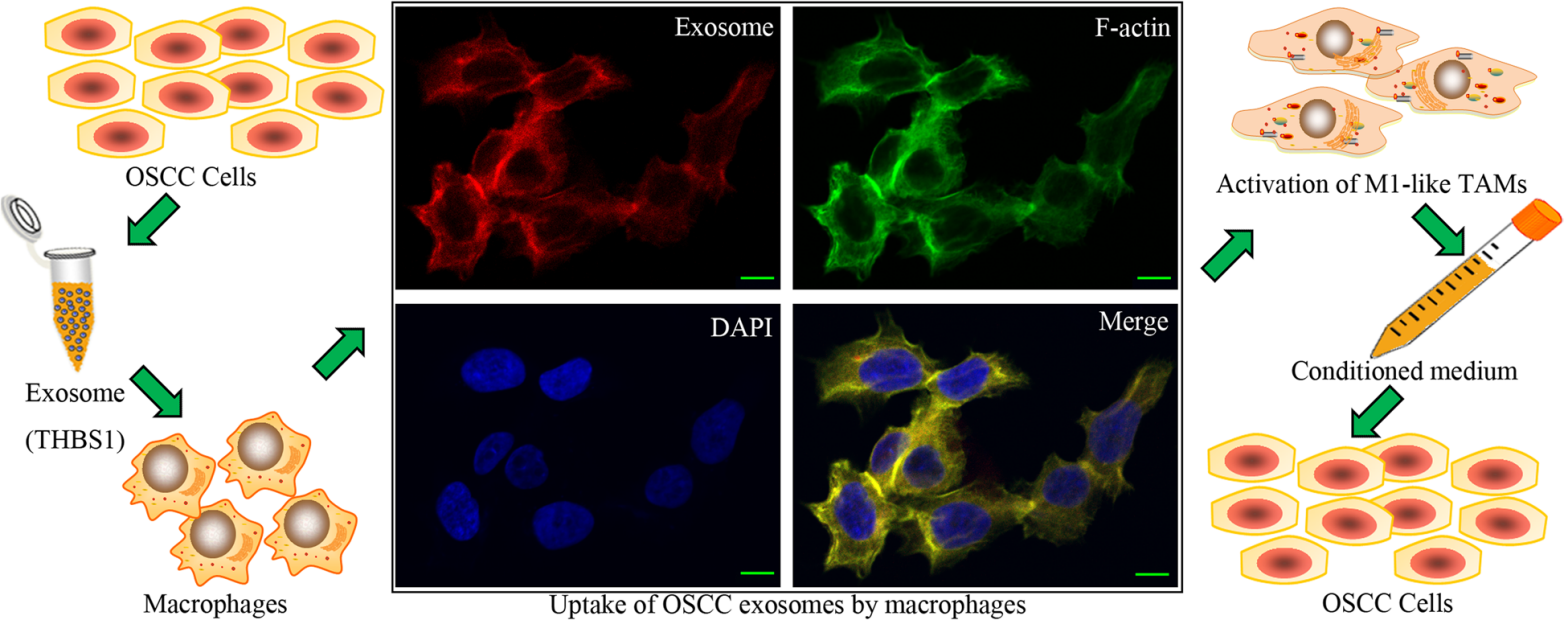
A schematic diagram for our previous study on the M1-like TAMs activated by exosome-transferred THBS1 in OSCC (bar=25μm), and for this study on the feedback roles of the M1-like TAMs in the malignant regulation of OSCC cells

**Fig. 2:**
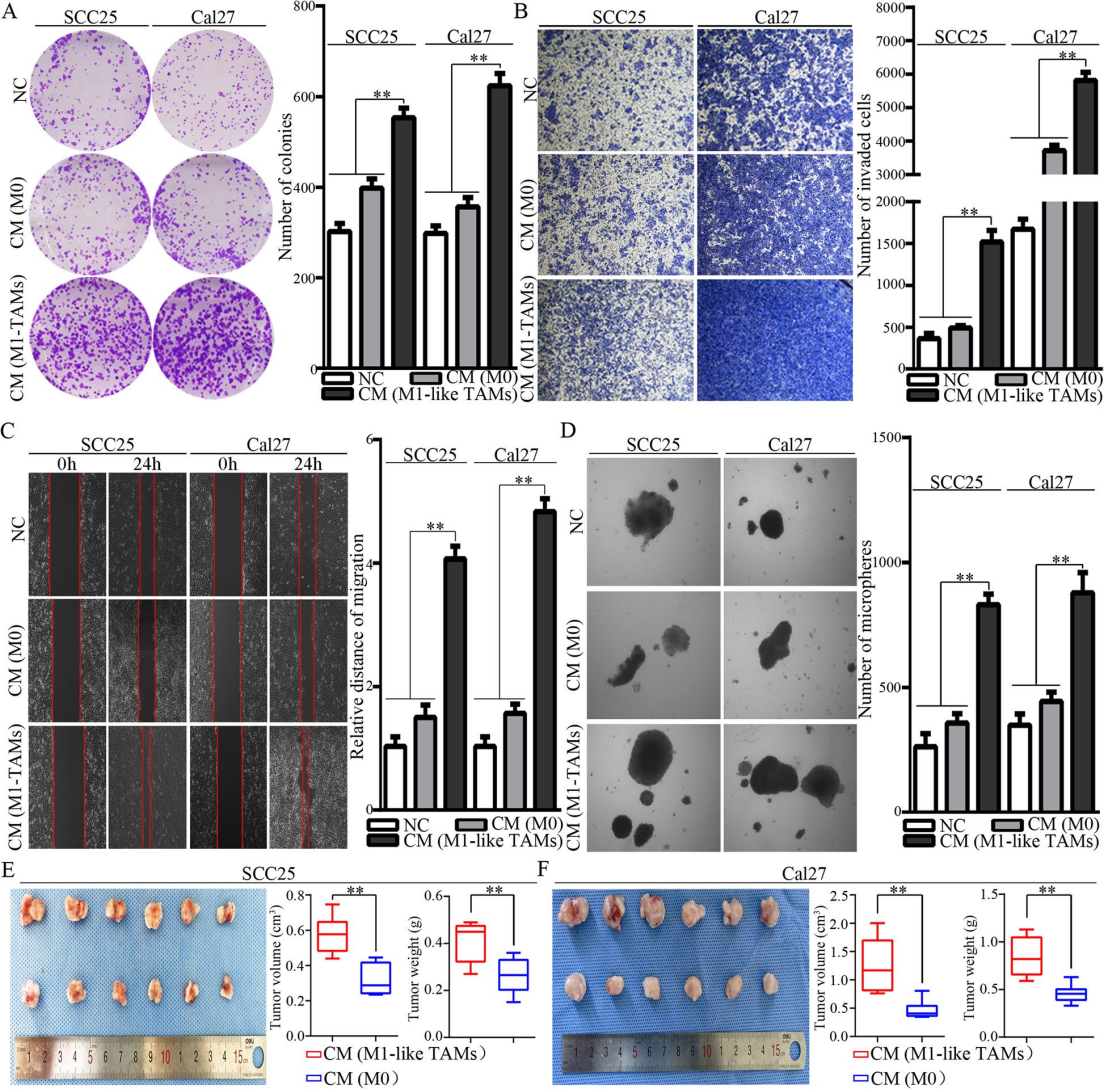
The effects of CM from M1-like TAMs on the malignant behaviors of OSCC cells. A-D. The effect of CM on the colony formation (**A**), invasion (**B**), migration (**C**), and microsphere forming (**D**) potential of OSCC cells; Data were represented as the mean ± SD of three independent experiments, **p<0.01. **E-F**. The effects of CM from M1-like TAMs on the malignant behaviors of OSCC cells in *vivo*. Mice with OSCC xenografts were received intraperitoneal injection of 1ml CM from M1-like TAMs or from M0 5 days per week, respectively. **E**. Representative images for SCC25 xenografts, and tumor volume and tumor weight were compared; **F**. Representative images for Cal27 xenografts, and tumor volume and tumor weight were compared, **p<0.01

We have identified a polarization of M1-like TAMs by exosome-transferred THBS1 from OSCC cells. To investigate the biological functions of M1-like TAMs in OSCC, conditioned media (CM) from M1-like TAMs were harvested and used to treat OSCC cells (Fig.  1). Comparatively, CM from M1-like TAMs significantly increased the colony formation of OSCC cells (Fig.  2A). In addition, CM from M1-like TAMs caused a dramatic increase in the invasion and migration abilities of OSCC cells (Fig.  2B, C). Microsphere forming assays indicated that CM from M1-like TAMs could significantly increase microsphere forming *in vitro* (Fig. 2D). Compared to CM from M0 cells, CM from M1-like TAMs greatly increased the tumor forming of OSCC xenografts *in vivo* (Fig. 2E).

### M1-like TAMs tightly correlate with the EMT process and CSCs characteristics of OSCC cells

**Fig. 3:**
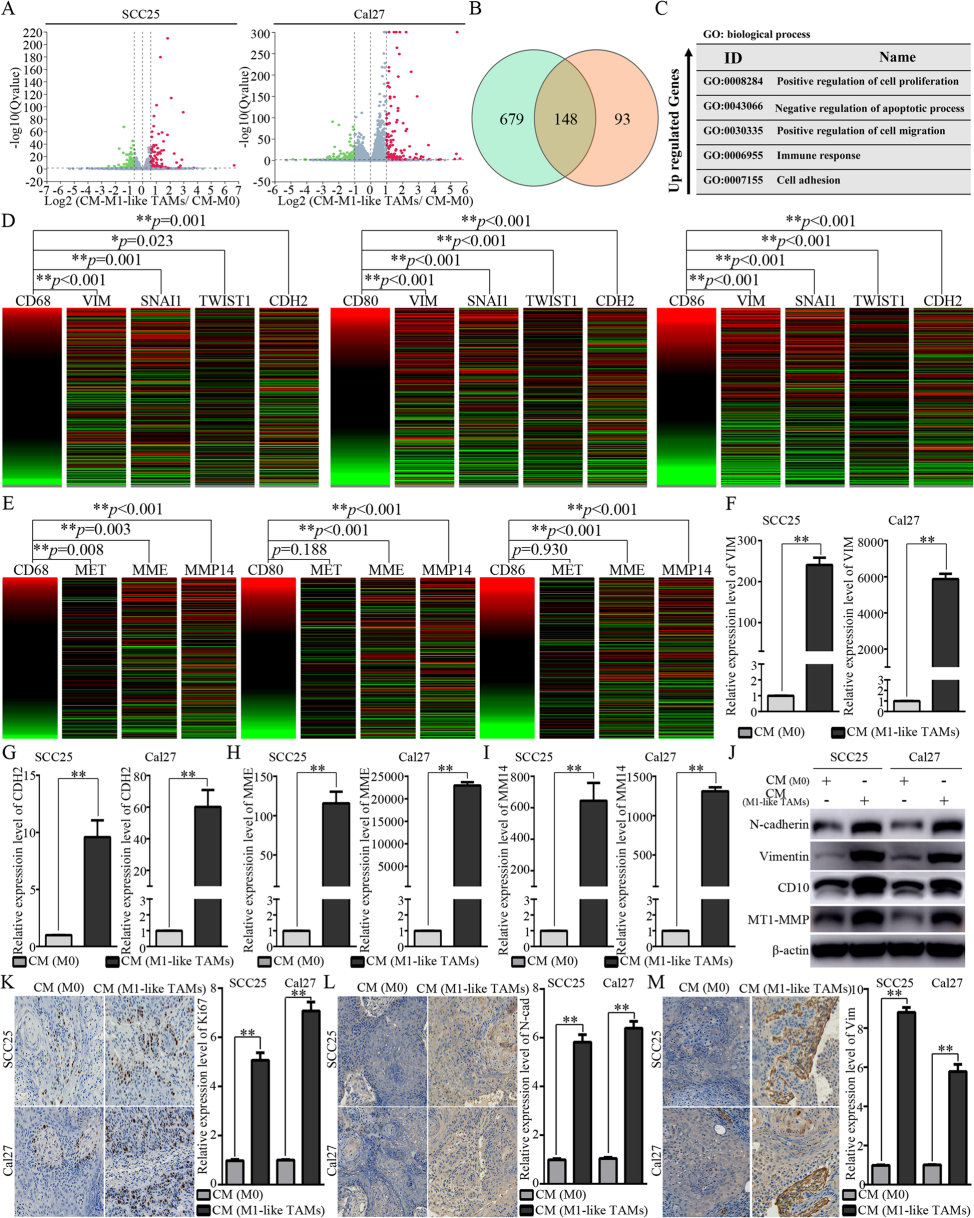
Significant correlations between M1-like TAMs and EMT/CSCs status of OSCC cells indicated by mRNA sequencing and further validation. **A**. Volcanic plots for the mRNA sequencing of SCC25 cells and Cal27 cells treated by CM from M1-like TAMs or M0 cells; **B**. Venn diagram indicating 148 differential expression genes shared by SCC25 cells and Cal27 cells; **C**. GO analysis for the up-regulated differential expression genes shared by SCC25 cells and Cal27 cells; **D**. Heat-map from the UCSC Xena Browser based on the TCGA primary HNSCC cohort depicted the gene expression relationship between M1 related markers (CD68, CD80, and CD86) and EMT related markers (VIM, SNAI1, TWIST1, and CDH2). **E**. Heat-map from the UCSC Xena Browser based on the TCGA primary HNSCC cohort depicted the gene expression relationship between M1 related markers (CD68, CD80, and CD86) and CSC related markers (MET, MME, and MMP14); **F-G**. Significantly increased expression of EMT related markers (VIM and CDH2) in OSCC cells treated by CM from M1-like TAMs for 4 days; **H-I**. Significantly increased expression of CSC related markers (MME and MMP14) in OSCC cells treated by CM from M1-like TAMs for 6 days; **J**. An increased expression of N-cadherin (encoded by CDH2), Vimentin (encoded by VIM), CD10 (encoded by MME), and MT1-MMP (encoded by MMP14) in OSCC cells treated by CM from M1-like TAMs for 7 days; **K-M**. Representative images and relative expression for the Ki67 (**K**), N-cadherin (**L**), and Vimentin (**M**) of the xenografts (200x); **p<0.01

After treatment with CM from M0 or M1-like TAMs, OSCC cells were harvested for further RNA sequencing analysis (Additional File 1A). The differentially expressed genes (DEGs) were filtered and identified (Fig. 3A, Additional File 1B-D). As shown in Fig. 3B, 148 DEGs were shared between SCC25 cells and Cal27 cells. GO analysis for the shared upregulated genes indicated that CM from M1-like TAMs could affect the cellular proliferation, apoptotic process, cell migration, immune response and cell adhesion of OSCC cells (Fig. 3C). Accordingly, we proposed that M1-like TAMs might promote the malignant progression of OSCC cells by regulating EMT/CSCs. Bioinformatics analyses indicated significant correlations between M1 related markers (CD68, CD80 and CD86) and EMT related markers (VIM, SNAI1, TWIST1 and CDH2) (Fig. 3D). Besides, significant correlations between M1-related markers and CSCs related markers (MME and MMP14) were also identified based on the TCGA primary HNSCC cohort (Fig. 3E). When the OSCC cells were treated with the CM from the M1-like TAMs, a sharp increase could be observed for EMT related markers (VIM and CDH2, Fig. 3F, G) and CSCs related markers (MME and MMP14, Fig. 3H, I). WB assays indicated a significantly increased expression of N-cadherin, Vimentin, CD10, and MT1-MMP in OSCC cells treated with CM from the M1-like TAMs (Fig. 3J). In OSCC xenografts, CM from M1-like TAMs induced significantly increased expression of Ki67 (Fig. 3K), N-cadherin (Fig. 3L) and Vimentin (Fig. 3M). The above information indicated that M1-like TAMs could regulate the EMT/CSC process of OSCC cells.

### IL6 secreted from M1-like TAMs promotes the malignant progression of OSCC cells

**Fig. 4:**
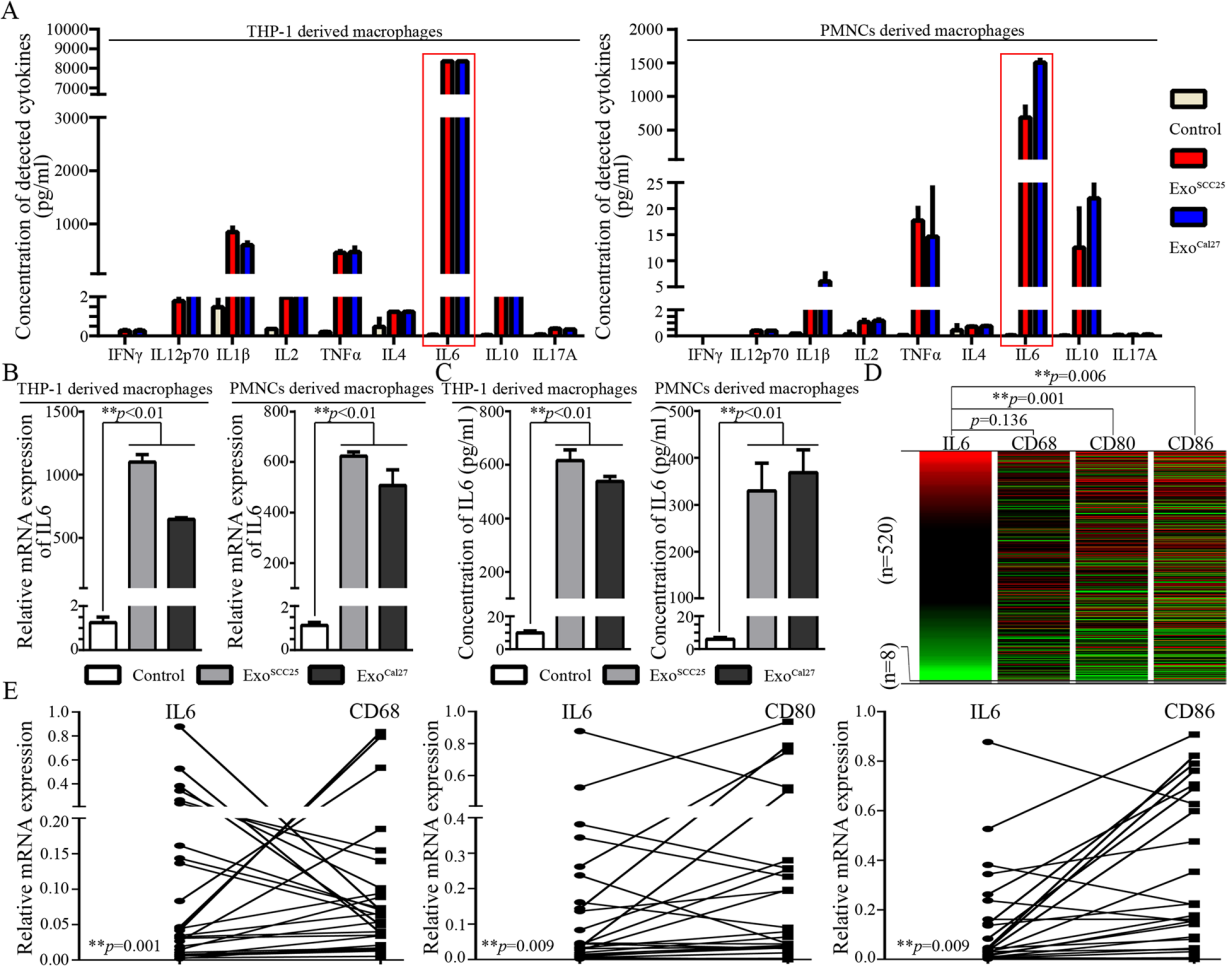
IL6 secreted from the M1-like TAMs activated by exosomes from OSCC cell lines. **A**. Cytokine expression profiles assessed by Luminex assay. THP-1 and PMNCs derived macrophages were treated with exosome supernatant from SCC25 and Cal27 for 24h, respectively; **B**. Validation for the mRNA expression of IL6 in the THP-1 and PMNCs derived macrophages treated with exosome supernatant from SCC25 and Cal27 for 12h; **C**. Validation for the IL6 secretion in the THP-1 and PMNCs derived macrophages treated with exosome supernatant from SCC25 and Cal27 for 24h; Data were represented as the mean ± SD of three independent experiments, **p<0.01. **D**. Heat-map from the UCSC Xena Browser based on the TCGA primary HNSCC cohort depicted the gene expression relationship between IL6 and CD68, CD80 or CD86; **E**. Expression pattern of IL6, CD68, CD86, and CD80 in a validated primary OSCC cohort (*n*=25). Crosstab analyses were performed for all the involved cases and Chi-squared tests were performed to assess the expression correlations between two variables **p<0.01

**Fig. 5:**
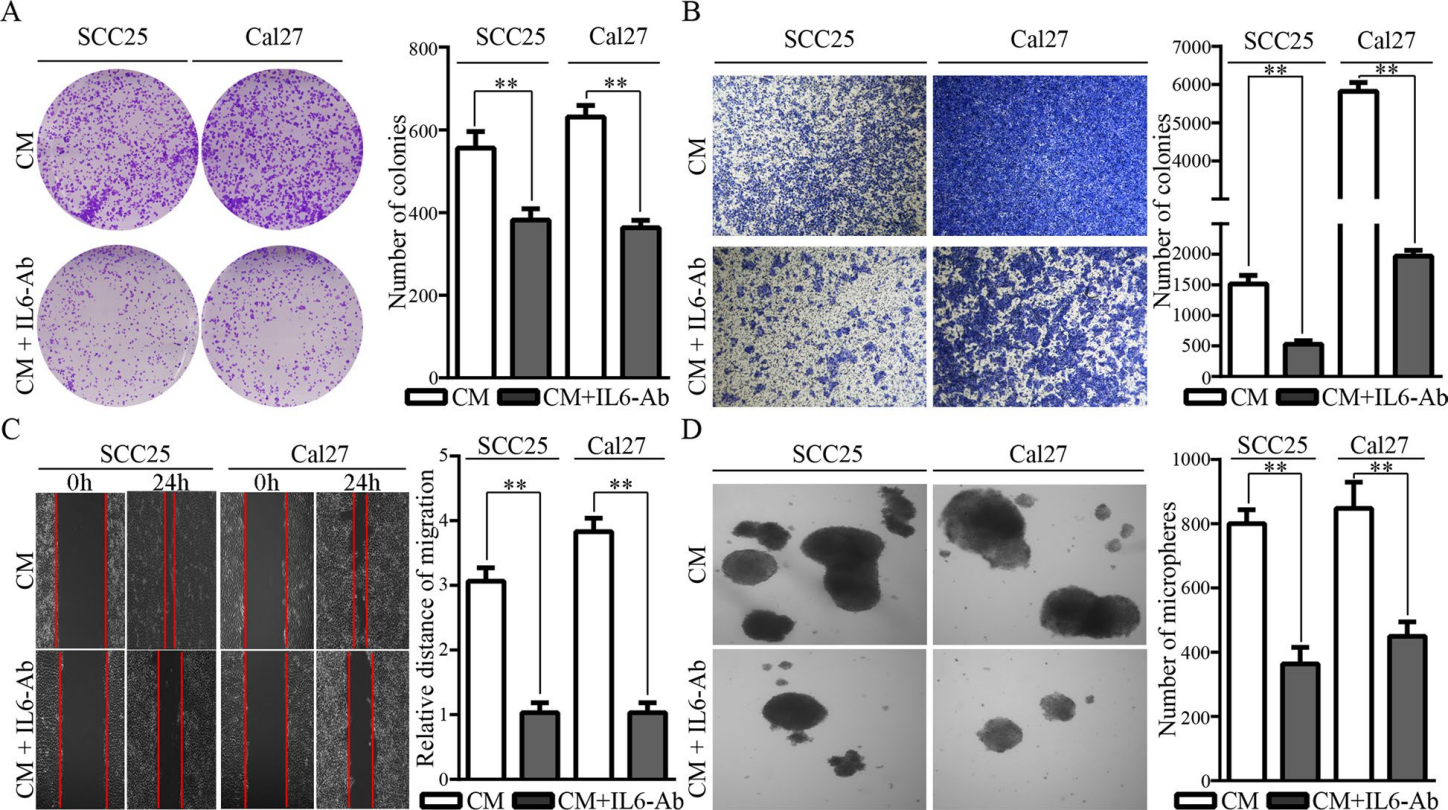
IL6-Ab reduced the effects of CM from M1-like TAMs on the malignant behaviors of OSCC cells in *vitro*. IL6-Ab reduced the effect of CM on the colony formation (**A**), invasion (**B**), migration (**C**), and microsphere forming (**D**) potential of OSCC cells. Data were represented as the mean ± SD of three independent experiments, **p<0.01

In this study, cytokine patterns for the CM of M1-like TAMs were investigated to identify the potential cytokines that might participate in promoting the malignant progression of OSCC cells. Consequently, a significant increase in IL6 expression was observed and validated in macrophages treated with exosomes from OSCC cells (Fig. 4A-C). Further bioinformatics analyses depicted a significant correlation between IL6 expression and the expression of M1 related markers (CD80 and CD86) (Fig. 4D). In the validated OSCC cohort, a significant correlation was also observed (Fig. 4E). Significant correlations between IL6 and EMT/CSCs have also been widely identified in other cancer types (Additional file 2). Then, we managed to block the IL6 in the CM of M1-like TAMs. As shown in Fig. 5A-D, IL6-Ab significantly blocked the effects of CM from M1-like TAMs on the colony forming, invasion, migration and microsphere forming abilities of OSCC cells.

### M1-like TAMs regulate the malignant behaviors of OSCC cells via the JAK/STAT3 pathway

**Fig. 6:**
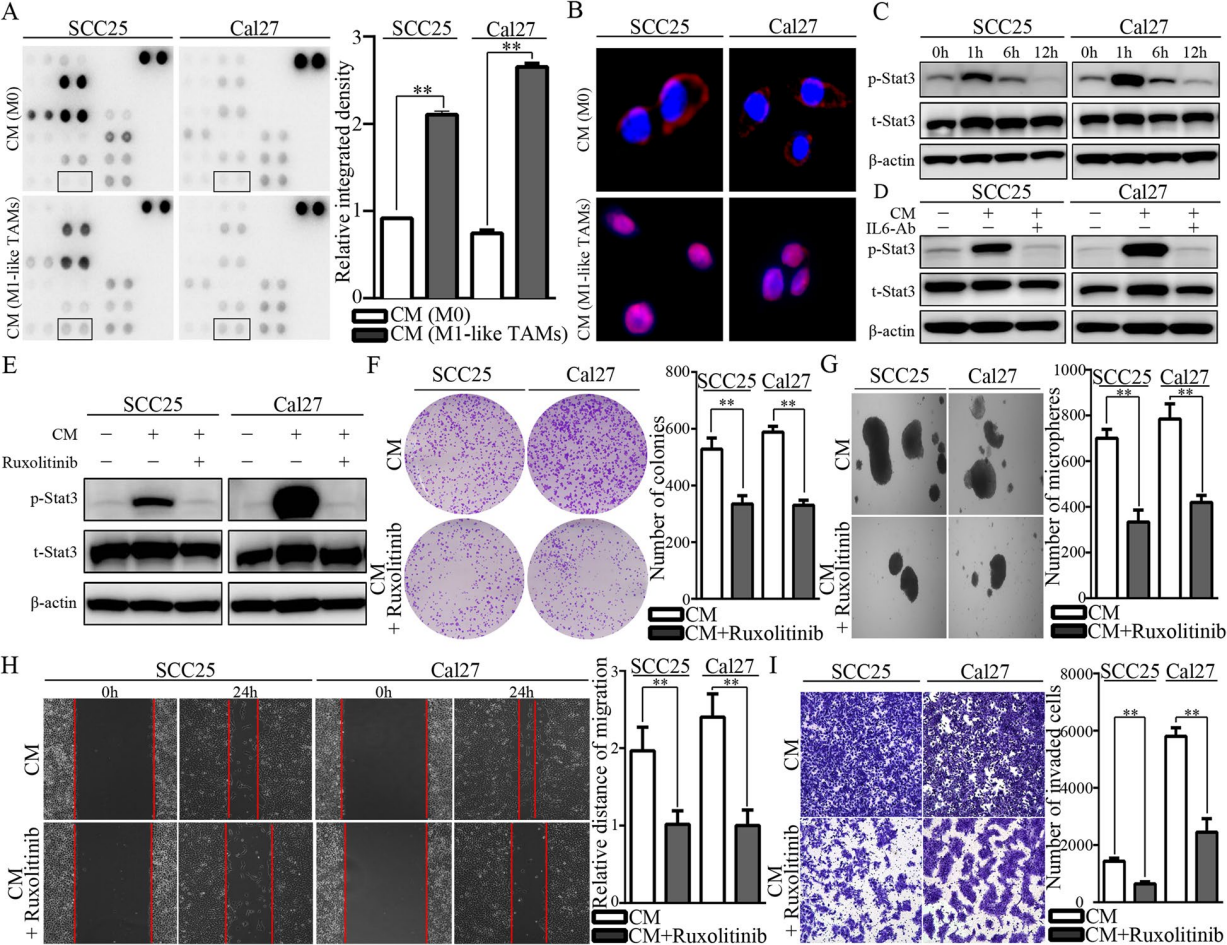
CM from M1-like TAMs regulated the malignant behaviors of OSCC cells via the JAK/STAT3 pathway. **A**. OSCCs cells were lysed after treatment with CM from M1-like TAMs or M0 for 6h, and the activation status of cellular signaling was detected by using Human Phospho-kinase Array Kit. A significant activation of the p-Stat3 (Y705) was detected in the SCC25 and Cal27 cells treated with CM from M1-like TAMs; **B**. The activation of Stat3 was validated by immunofluorescence in SCC25 and Cal27 cells treated with CM from M1-like TAMs (400x); **C**. The activation of Stat3 was validated by WB in SCC25 and Cal27 cells treated with CM from M1-like TAMs in a time-dependent manner; **D**. IL6-Ab blocked the activation of Stat3 in SCC25 and Cal27 cells treated with CM from M1-like TAMs; **E**. The activation of Stat3 in SCC25 and Cal27 cells were blocked under the treatment with CM from M1-like TAMs and Ruxolitinib (50nM). **F-I**. Ruxolitinib (50nM) reduced the effects of CM from M1-like TAMs on the colony formation (**F**), microsphere forming (**G**), migration (**H**), and invasion (**I**) potential of SCC25 and Cal27 cells. Data were represented as the mean ± SD of three independent experiments, **p<0.01

**Fig. 7:**
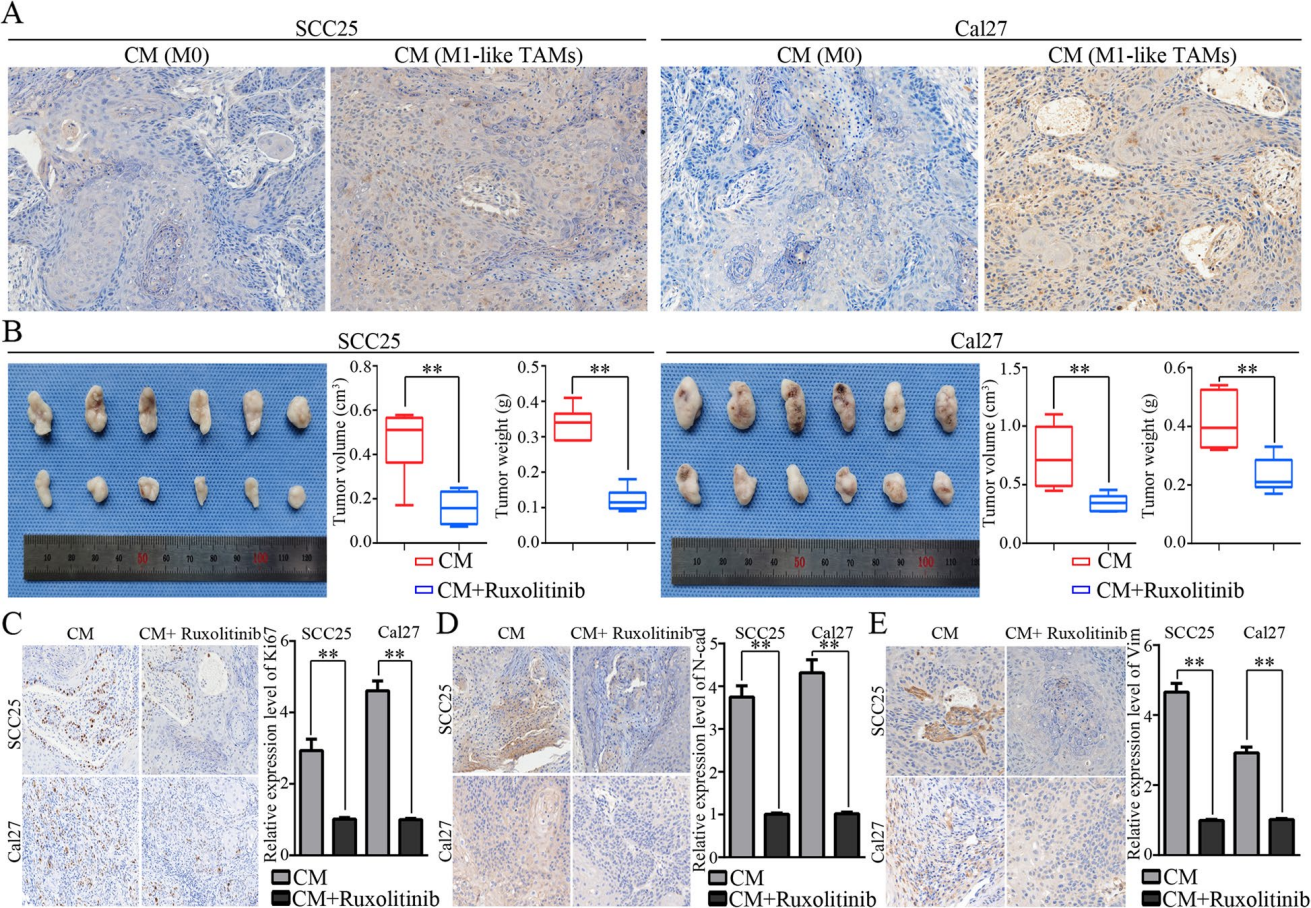
An in *vivo* validation for the activation of the JAK/STAT3 pathways by CM from M1-like TAMs. **A**. An increased expression of p-Stat3 in the OSCC xenografts treated by CM from M1-like TAMs (200x); **B**. Ruxolitinib (50 mg/kg, i.p. every other day) significant reduced the tumor forming of OSCC cells treated with CM from M1-like TAMs; **C-E.** Ruxolitinib significant reduced the expression of Ki67 (**C**), N-cadherin (**D**), and Vimentin (**E**) in the OSCC xenografts treated by CM from M1-like TAMs (200x); **p< 0.01

Then, we investigated the underlying mechanism by which M1-like TAMs achieve the regulation of OSCC cells. The activation status of cellular signaling pathways in OSCC cells was detected during the treatment of CM from M1-like TAMs. Significantly increased activation of p-Stat3 (Y705) was detected in SCC25 and Cal27 cells treated with CM from M1-like TAMs (Fig. 6A-C). In addition, IL6-Ab blocked the activation of p-Stat3 in OSCC cells treated with CM from M1-like TAMs (Fig. 6D). The Jak/STAT3 pathway is a classical signaling pathway downstream of IL6 activation. A Jak inhibitor (ruxolitinib) effectively inhibited the activation of Stat3 signaling in OSCC cells treated with CM from M1-like TAMs (Fig. 6E). As shown in Fig. 6F-I, ruxolitinib significantly blocked the effects of CM from M1-like TAMs on the colony forming, microsphere forming, migration, and invasion abilities of OSCC cells *in vitro*. In situ activation of p-Stat3 was also observed in OSCC xenografts treated with CM from M1-like TAMs (Fig.  7A). Moreover, ruxolitinib successfully inhibited the tumor forming of OSCC xenografts treated by CM from M1-like TAMs *in vivo* (Fig. 7B). Histopathologically, decreased expression of Ki67, N-cadherin and Vimentin could be observed in OSCC xenografts treated with CM from M1-like TAMs and ruxolitinib (Fig. 7C-E). The above data indicated that M1-like TAMs could regulate the EMT/CSCs characteristics of OSCC cells via the IL6/Jak/Stat3 pathway.

### M1-like TAMs cascade a mesenchymal/stem-like phenotype of OSCC cells through the IL6/Jak/Stat3/THBS1 axis

**Fig. 8:**
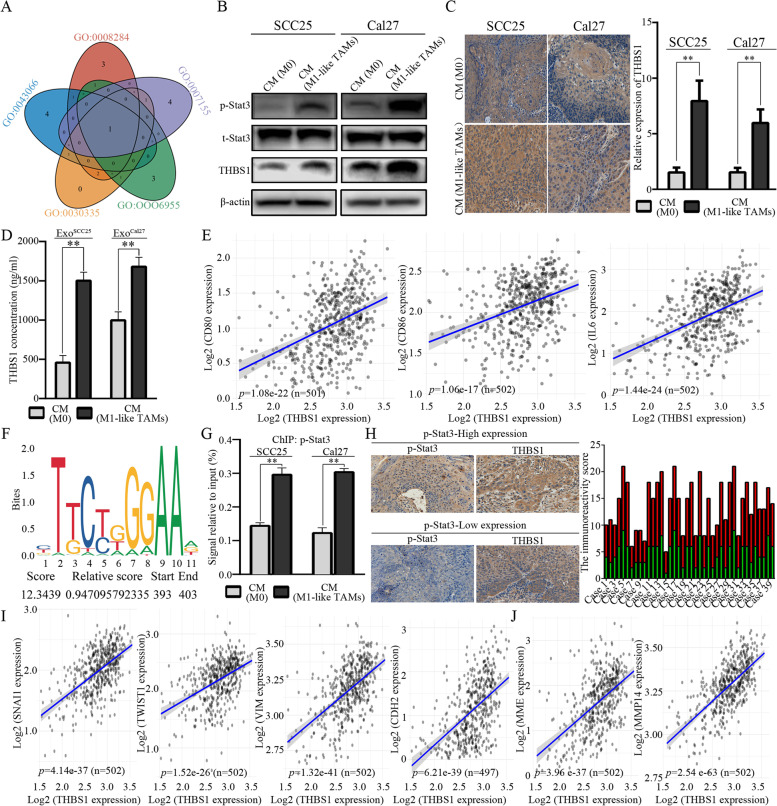
M1-like TAMs cascade the EMT/CSCs regulation of OSCC cells through the Jak/Stat3/THBS1 pathway. **A**. Venn diagram for the up-regulated differential expression genes shared by each filtered GO analysis; **B**. An increased expression of THBS1 by the activation of Stat3 in OSCC cells treated with CM from M1-like TAMs; **C**. Increased expression of THBS1 in the OSCC xenografts treated with CM from M1-like TAMs (200x); **D**. Increased expression of THBS1 in the exosomes derived from OSCC cells treated with CM from M1-like TAMs; **E**. Bioinformatics analyses indicating a significantly positive correlation between THBS1 expression and CD80/CD86/IL6 expression in the TCGA HNSCC cohort; **F**. Promoter prediction indicating the expression of THBS1 targeted by Stat3; **G.** ChIP assays were performed in OSCC cells after treatment with CM from M0 and M1-like TAMs for 30min; **H.** The correlation between STAT3 activation and THBS1 expression in OSCC samples, the immunoreactivity scores were recorded by multiplying the staining intensity and the percentage of positive cancer cells (*n*=40, 200x, *p*< 0.01); **I**. Bioinformatics analyses for the expression correlation between THBS1 and EMT related genes based on the TCGA primary HNSCC cohort; **J.** Bioinformatics analyses for the expression correlation between THBS1 and CSC related genes based on the TCGA primary HNSCC cohort

Venn analysis was performed to filter the up-regulated DEGs shared by each filtered GO analysis in OSCC cells treated with CM from M1-like TAMs (Fig. 8A, Additional file 3A). Accordingly, THBS1 was filtered as a candidate target transcribed by Stat3. In OSCC cells treated with CM from M1-like TAMs, the activation of Stat3 resulted in increased expression of THBS1 (Fig. 8B). Increased expression of THBS1 was also observed in situ in OSCC xenografts treated with CM from M1-like TAMs (Fig. 8C). What’s more, CM from M1-like TAMs could significantly increase the expression level of THBS1 in the exosomes derived the treated OSCC cells (Fig. 8D). Bioinformatics analysis indicated significant correlations between THBS1 and CD80/CD86/IL6 (Fig. 8E). Promoter prediction analysis was conducted to validate the relationship between transcription factor Stat3 and the filtered DEGs. The highest score was observed for THBS1 (Fig. 8F, Additional file 3B). ChIP assays identified that CM from M1-like TAMs could effectively promote the enrichment of p-Stat3 in promoter regions of THBS1 (Fig. 8G). In the validated OSCC samples, we also observed increased expression of THBS1 in the sample with increased activation of p-Stat3 (Fig. 8H). Furthermore, significant correlations were observed between THBS1 and EMT/CSC-related markers (Snai1, Twist1, Vimentin and Cadherin, Fig. 8I; MME and MMP14, Fig. 8J). Increased expression of THBS1 could significantly decrease the overall survival (OS) of HSNCC patients (Additional file 4). The above information indicated that M1-like TAMs could regulate the EMT/CSCs process of OSCC cells through the IL6/Jak/Stat3 signaling, which could subsequently promote the transcription and expression of THBS1.

## Discussion

**Fig. 9:**
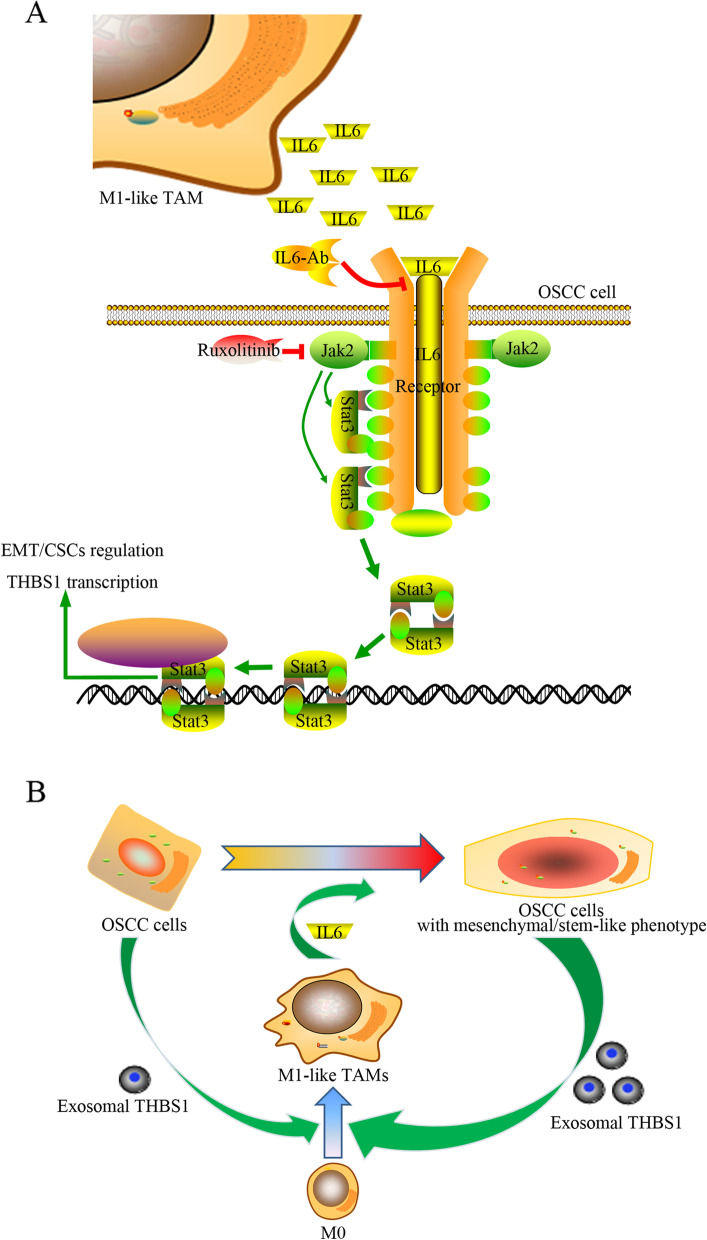
Schematic diagrams depict that M1-like TAMs activated by exosome-THBS1 regulate the EMT/CSC cascades of OSCC cells by the IL6/STAT3/THBS1 axis. **A**. IL6 released by M1-like TAMs activates the STAT3 signaling in OSCC cells; **B**. A novel malignant loop demonstrates M1-like TAMs cascade a mesenchymal/stem-like phenotype of OSCC cells through the IL6/Jak/Stat3/THBS1 axis

The initiation of CSCs and the acquisition of EMT are key events for the malignant progression of OSCC, which also pose great challenges for the treatment outcomes of OSCC patients [18, 19]. A number of studies have focused on the genetic alterations that might be responsible for the initiation and progression of OSCC; however, no exact genetic events have been definitively validated [20, 21]. Meanwhile, immunological disorders in the TME of OSCC have been investigated and acknowledged [14, 22]. TAMs are one of the major types of tumor infiltrating innate immune cells, and have been described as crucial drivers of tumor-promoting inflammation in various cancers [2]. Previously, we have identified and reported a novel paracrine loop between OSCC and M1-like TAMs via exosome-transferred THBS1 [8]. In this study, we managed to demonstrate the roles of M1-like TAMs in the malignant progression of OSCC cells in detail. We observed that abundant IL6 was secreted by M1-like TAMs activated by exosomes from OSCC cells. Activated M1-like TAMs led to a mesenchymal/stem-like phenotype of OSCC via IL6/Stat3 pathways (Fig. 9A). In addition, the activation of STAT3 increased the transcription of THBS1 in OSCC cells, which cascaded an enhanced feedback loop between M1-like TAMs and OSCC cells with a mesenchymal/stem-like phenotype (Fig. 9B).

Epithelial-mesenchymal transition is a process in which epithelial tumor cells lose their epithelial and gain mesenchymal function [18]. EMT induces cancer cell movement, cancer progression, metastasis and stemnes s [23]. The upregulation of CSC-related transcription factors (Oct4, Sox2, Nanog) has been identified in OSCC EMT [18]. Accordingly, TAMs are the most important ancillary cells regulating the EMT process and CSC activities in various cancers [4, 24, 25]. Studies have shown that TAMs are able to induce EMT/CSCs through various signaling pathways such as the TLR4/IL10 pathways, the TGF-β/Smad2 pathway, the miR-30a/NF-κB/Snail signaling pathways, Wnt2b/β-catenin/c-Myc signaling pathways, Stat3 pathways, and etc [3, 4, 25, 26]. After filtered all the reported CSCs-related markers in OSCC [27], we detected an increased expression of MME and MMP14 in OSCC cells treated with CM from M1-like TAMs. In this study, we demonstrated for the first time that the activated M1-like TAMs could significantly promote the CSCs process via up-regulating the expression of MME and MMP14 in OSCC. Previously, it was reported that inflammatory factors secreted by TAMs with the M1 phenotype could induce breast cancer cells to undergo a partial EMT [28]. Although the roles of M1-like TAMs in cancer progression have been reported, the underlying mechanisms are poorly understood. Herein, cytokine patterns from activated M1-like TAMs were investigated in detail, and a sharp increase in IL6 secretion was observed and validated. We found that IL6 secreted from the activated M1-like TAMs participated in promoting the malignant process of OSCC by regulating EMT and the induction of CSCs.

Interleukin 6 has been demonstrated to be a pleiotropic cytokine in the regulation of cancer progression, and it is involved in EMT, CSCs, angiogenesis, and therapeutic resistance [29, 30]. In this study, we demonstrated that exosome-activated M1-like TAMs could be an important origin of IL6 in the TME of OSCC. Moreover, the IL6/Jak/Stat3 signaling participated in the regulation of EMT/CSCs in OSCC by activated M1-like TAMs. The Jak/Stat3 signaling axis is the classic downstream pathway activated by IL6 [29]. Stat3 is a transcription factor with many biological functions, and the hyper-activation of Stat3 widely regulates the malignant progression of cancer cells [31, 32]. In OSCC, the activation of the Jak/Stat3 signaling pathway has been shown to be able to promote the process of EMT and enhance the stemness of cancer cells [33, 34]. Above all, we demonstrated the roles of M1-like TAMs in promoting the process of EMT and enhancing CSCs in OSCC via the IL6/Jak/Stat3 signaling pathway.

We attempted to analyze the downstream targets of Stat3 activated by IL6 from M1-like TAMs, and a tight correlation between Stat3 and THBS1 was filtered. Previously, we identified that OSCC cells could polarize macrophages into M1-like TAMs by releasing exosomal THBS1 [8]. Herein, we observed and validated that the activation of M1-like TAMs promoted the expression of THBS1 in OSCC cells via IL6/Jak/Stat3 signaling. Consequently, more exosomal THBS1 could be released from transformed OSCC cells with the EMT/CSC phenotype, and more M1-like TAMs would be polarized by exosomal THBS1 from OSCC cells. Overall, a positive feedback loop was demonstrated between M1-like TAMs and OSCC cells in the regulation of EMT and CSCs, which might facilitate the development of novel therapies for improving the treatment of OSCC patients.

## Conclusions

In conclusion, we reveal a better understanding of the functional roles and associated molecular mechanisms of M1-like TAMs in regulating the malignant progression of OSCC. Targeting M1-like TAMs might be a great supplementary strategy for the current treatment of OSCC patients. Additional substantial and preclinical research is needed to validate the effectiveness and applicability of treating strategies targeting M1-like TAMs.

## Supplementary Information

**Additional file 1.**

**Additional file 2.**

**Additional file 3.**

**Additional file 4.**

## Data Availability

The datasets used and/or analyzed during the current study are available from the corresponding authors on reasonable request.

## Abbreviations

TAMs: Tumor-associated macrophages
OSCC: Oral squamous cell carcinoma
CM: Conditioned Media
EMT: Epithelial-mesenchymal transition
CSCs: Cancer-stem like cells
TME: tumor microenvironment
HNSCC: Head and neck squamous cell carcinoma
ATCC: American Type Culture Collection
PBMCs: Peripheral blood mononuclear cells
ELISA: Enzyme-linked Immunosorbent Assays
qRT-PCR: Quantitative real-time PCR
IHC: Immunohistochemical Staining
WB: Western blotting
ChIP: Chromatin immunoprecipitation
IL6: Interleukin 6
