# Supplementary material for: M1-like tumor-associated macrophages cascade a mesenchymal/stem-like phenotype of oral squamous cell carcinoma via the IL6/Stat3/THBS1 feedback loop
Source: J Exp Clin Cancer Res. 2022 Jan 6;41:10. doi: 10.1186/s13046-021-02222-z (PMC8734049; doi:10.1186/s13046-021-02222-z)
Supplement: Supplementary file 1 — Additional file 1. [file 13046_2021_2222_MOESM1_ESM.docx]

**Additional file 1**

**
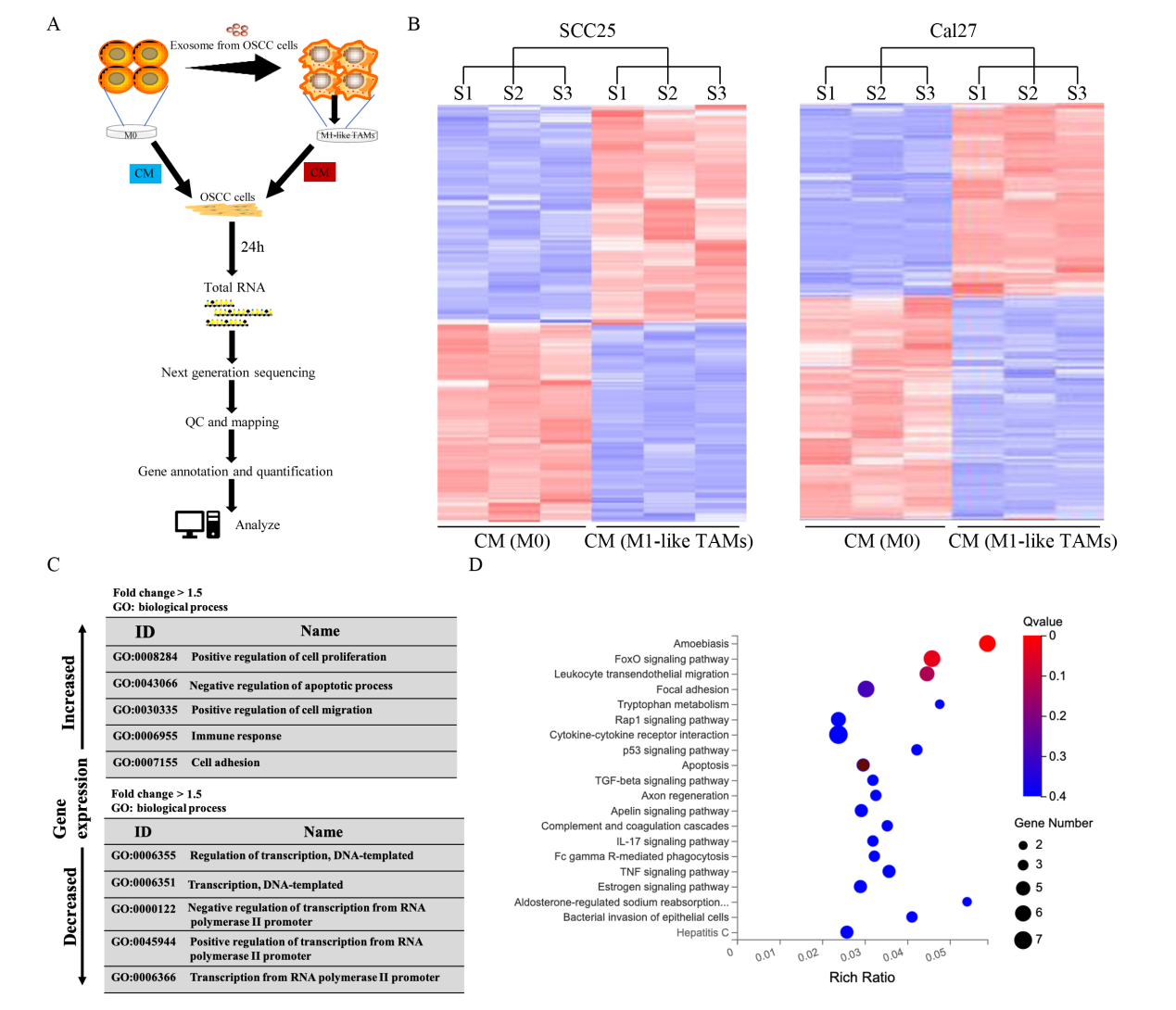
**

Additional file 1: A：The workflow for the mRNA sequencing of SCC25 cells and Cal27 cells treated by CM from M1-like TAMs or M0 cells; B: The heat-maps for the mRNA sequencing of SCC25 cells and Cal27 cells treated by CM from M1-like TAMs or M0 cells; three independent experiments were performed; C: GO analysis for the down-regulated differential expression genes shared by SCC25 cells and Cal27 cells; D: KEGG analysis for the differential expression genes shared by SCC25 cells and Cal27 cells.
