# Supplementary material for: M1-like tumor-associated macrophages cascade a mesenchymal/stem-like phenotype of oral squamous cell carcinoma via the IL6/Stat3/THBS1 feedback loop
Source: J Exp Clin Cancer Res. 2022 Jan 6;41:10. doi: 10.1186/s13046-021-02222-z (PMC8734049; doi:10.1186/s13046-021-02222-z)
Supplement: Supplementary file 2 — Additional file 2. [file 13046_2021_2222_MOESM2_ESM.docx]

**Additional file 2**

**
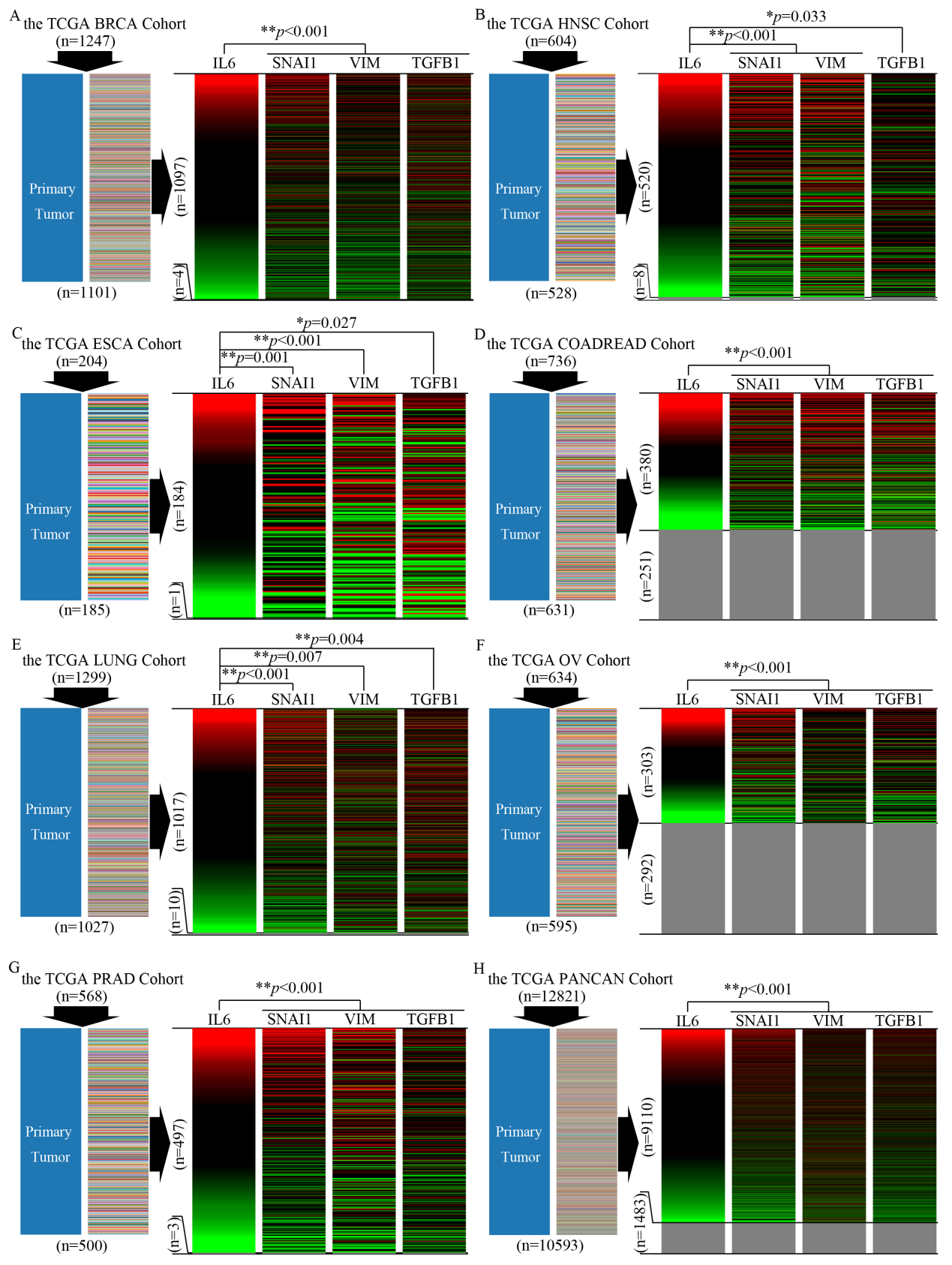
**

Additional file 2: The expression relationship between IL6 and EMT related molecules among various cancers. A-H:Heat-map from the UCSC Xena Browser based on the TCGA BRCA, HNSC, ESCA, COADREAD, LUNG, OV, PRAD, and PANCAN cohort depicts the gene expression relationship between IL6 and SANIL, VIM, TGFβ1. **p*<0.05, ***p*<0.01.
