# Supplementary material for: M1-like tumor-associated macrophages cascade a mesenchymal/stem-like phenotype of oral squamous cell carcinoma via the IL6/Stat3/THBS1 feedback loop
Source: J Exp Clin Cancer Res. 2022 Jan 6;41:10. doi: 10.1186/s13046-021-02222-z (PMC8734049; doi:10.1186/s13046-021-02222-z)
Supplement: Supplementary file 3 — Additional file 3. [file 13046_2021_2222_MOESM3_ESM.docx]

**Additional file 3**

**
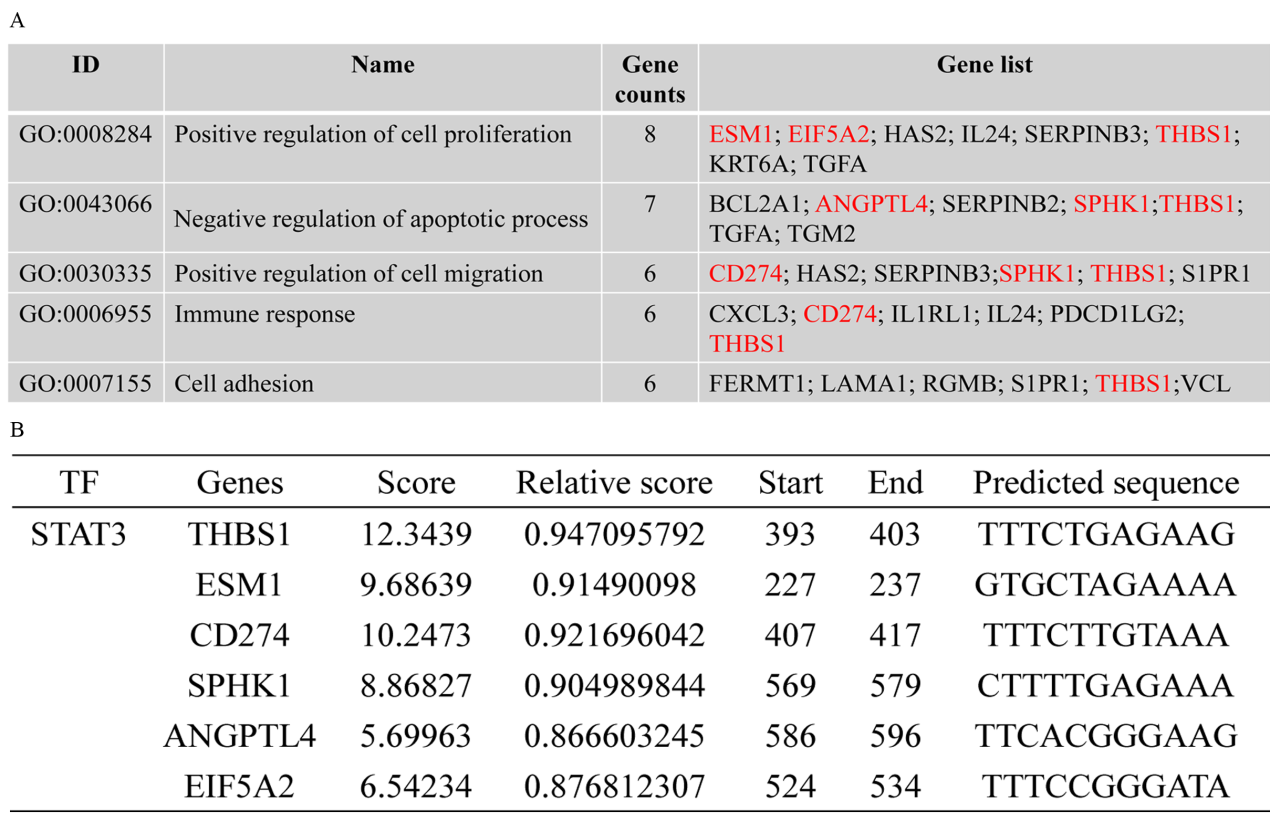
**

Additional file 3: A: GO analysis and the involved genes for the up-regulated differential expression genes shared by SCC25 cells and Cal27 cells; B: Promoter prediction for all the filtered genes indicated by GO analysis.
