# Supplementary material for: M1-like tumor-associated macrophages cascade a mesenchymal/stem-like phenotype of oral squamous cell carcinoma via the IL6/Stat3/THBS1 feedback loop
Source: J Exp Clin Cancer Res. 2022 Jan 6;41:10. doi: 10.1186/s13046-021-02222-z (PMC8734049; doi:10.1186/s13046-021-02222-z)
Supplement: Supplementary file 4 — Additional file 4. [file 13046_2021_2222_MOESM4_ESM.docx]

**Additional file 4**

**
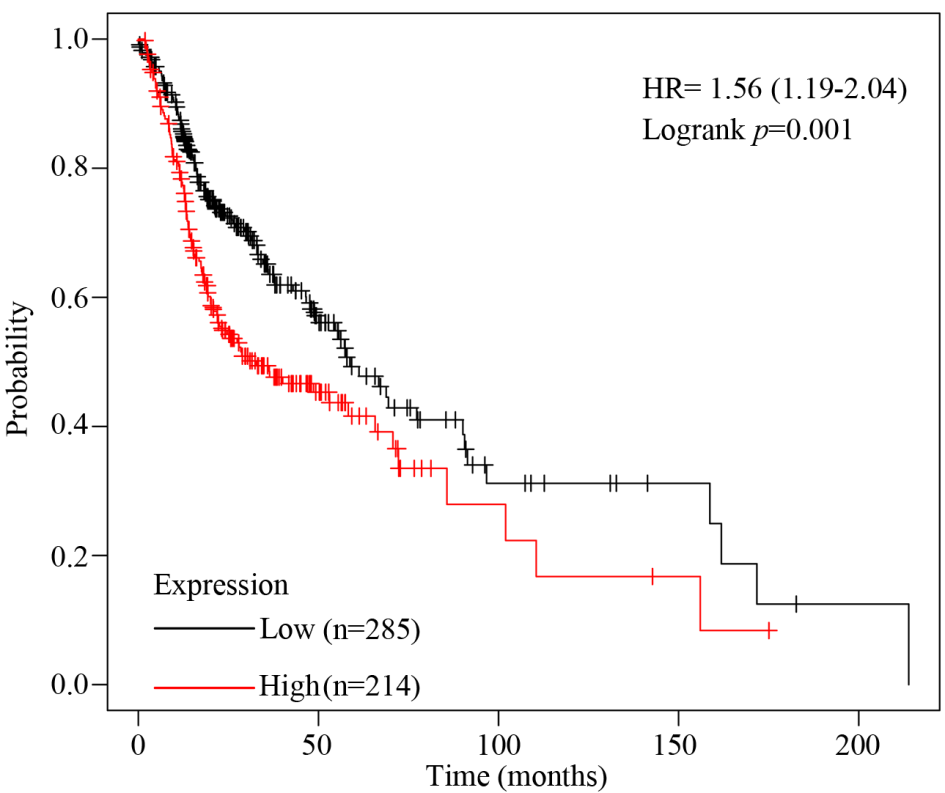
**

Additional file 4: Prognosis analysis for THBS1 in patients with HNSCC by using Kaplan-Meier Plotter.
